# Optical Coherence Tomography Reveals Distinct Patterns of Retinal Damage in Neuromyelitis Optica and Multiple Sclerosis

**DOI:** 10.1371/journal.pone.0066151

**Published:** 2013-06-21

**Authors:** Elisa Schneider, Hanna Zimmermann, Timm Oberwahrenbrock, Falko Kaufhold, Ella Maria Kadas, Axel Petzold, Frieder Bilger, Nadja Borisow, Sven Jarius, Brigitte Wildemann, Klemens Ruprecht, Alexander U. Brandt, Friedemann Paul

**Affiliations:** 1 NeuroCure Clinical Research Center, Charité - Universitätsmedizin Berlin, Berlin, Germany; 2 MS Centre Amsterdam, VU University Medical Centre, Amsterdam, The Netherlands; 3 Division of Molecular Neuroimmunology, Department of Neurology, University of Heidelberg, Heidelberg, Germany; 4 Clinical and Experimental Multiple Sclerosis Research Center, Charité - Universitätsmedizin Berlin, Berlin, Germany; 5 Department of Neurology, Charité - Universitätsmedizin Berlin, Berlin, Germany; 6 Max Delbrück Center for Molecular Medicine, Berlin, Germany; Friedrich-Alexander University Erlangen, Germany

## Abstract

**Background:**

Neuromyelitis optica (NMO) and relapsing-remitting multiple sclerosis (RRMS) are difficult to differentiate solely on clinical grounds. Optical coherence tomography (OCT) studies investigating retinal changes in both diseases focused primarily on the retinal nerve fiber layer (RNFL) while rare data are available on deeper intra-retinal layers.

**Objective:**

To detect different patterns of intra-retinal layer alterations in patients with NMO spectrum disorders (NMOSD) and RRMS with focus on the influence of a previous optic neuritis (ON).

**Methods:**

We applied spectral-domain OCT in eyes of NMOSD patients and compared them to matched RRMS patients and healthy controls (HC). Semi-automatic intra-retinal layer segmentation was used to quantify intra-retinal layer thicknesses. In a subgroup low contrast visual acuity (LCVA) was assessed.

**Results:**

NMOSD-, MS- and HC-groups, each comprising 17 subjects, were included in analysis. RNFL thickness was more severely reduced in NMOSD compared to MS following ON. In MS-ON eyes, RNFL thinning showed a clear temporal preponderance, whereas in NMOSD-ON eyes RNFL was more evenly reduced, resulting in a significantly lower ratio of the nasal versus temporal RNFL thickness. In comparison to HC, ganglion cell layer thickness was stronger reduced in NMOSD-ON than in MS-ON, accompanied by a more severe impairment of LCVA. The inner nuclear layer and the outer retinal layers were thicker in NMOSD-ON patients compared to NMOSD without ON and HC eyes while these differences were primarily driven by microcystic macular edema.

**Conclusion:**

Our study supports previous findings that ON in NMOSD leads to more pronounced retinal thinning and visual function impairment than in RRMS. The different retinal damage patterns in NMOSD versus RRMS support the current notion of distinct pathomechanisms of both conditions. However, OCT is still insufficient to help with the clinically relevant differentiation of both conditions in an individual patient.

## Introduction

Neuromyelitis optica (NMO) is a rare autoimmune central nervous system (CNS) condition that predominantly affects the optic nerves and the spinal cord [1], [2]. While NMO had previously been regarded as variant of multiple sclerosis (MS), the recent detection of a highly specific serum biomarker for NMO, antibodies to the most abundant astrocytic CNS water channel aquaporin-4 (AQP4), as well as histopathological data has made clear that NMO is a condition distinct from MS [1], [3]–[7].

An early and accurate diagnosis of NMO and distinction from MS is crucial as prognosis is usually worse than in MS and treatment options for both diseases differ considerably [8]. Although the broad availability of commercial testing for antibodies to AQP4 has facilitated differentiation of NMO from MS, a correct diagnosis remains challenging, in particular in those NMO patients who test negative for AQP4 antibodies. Thus, a considerable number of patients are still misdiagnosed with MS [1], [9].

Optical coherence tomography (OCT) is a non-invasive interferometry technique that has been used to measure retinal nerve fiber layer (RNFL) thickness, total macular volume (TMV) and ganglion cell layer (GCL) thickness in MS and other neurological diseases [10]–[14]. In MS, numerous studies have consistently shown reduced RNFL, TMV, and GCL across disease subtypes and in both eyes with and without prior history of optic neuritis (ON) [14]–[22]. Moreover, measures of retinal neuroaxonal damage were found to correlate with brain atrophy in MS [23]–[27].

Previous OCT studies have shown that ON in NMO causes more severe neuronal damage and greater thinning of the RNFL than ON in MS [28]–[34], which is in line with the clinical experience that visual acuity in NMO is usually more severely affected and visual outcome poorer than in MS [2], [8]. Moreover, and again in contrast to MS, increase in retinal damage as measured by OCT seems to be exclusively linked to clinical ON attacks in NMO while progressive retinal neuroaxonal damage independent of clinically apparent ON as in MS is rare in NMO [35].

In NMO, there are only few studies on retinal OCT data acquired with the high-resolution spectral-domain (SD) technology [36]–[39] most of which did not present data on segmentation of deeper retinal layers. The goal of our study was to analyze OCT measures from all retinal layers in patients with NMO or MS rigorously matched for history of ON and in healthy controls (HC), and to relate these measures to visual functions. We were interested whether the more severe retinal affection in NMO-ON as compared to MS-ON described in earlier OCT studies would also be detectable in deeper retinal layers and whether damage patterns differ between both conditions. If so, OCT could be an additional tool to aid in the clinically challenging differential diagnosis of MS and NMO.

## Materials and Methods

### Study Participants

Seventeen patients with NMO and NMO spectrum disorder (NMOSD) [5] were prospectively recruited from the outpatient clinics of the NeuroCure Clinical Research Center and of the Department of Neurology, Charité Universitaetsmedizin Berlin. Thirteen patients fulfilled the 2006 diagnostic criteria for NMO, and 4 had NMOSD (3 with longitudinally extensive transverse myelitis (LETM), 1 with recurrent optic neuritis) with AQP4 antibodies [40]. Ninety four percent (16/17) were seropositive for AQP4 antibodies [41]–[43]. HC and relapsing-remitting MS (RRMS) patients according to the 2010 revised McDonald criteria [44] were matched from the center’s research database according to age and gender. NMOSD and MS patients were additionally matched eye-wise for history of ON, determined by medical record review. Exclusion criteria were eye or retina diseases other than ON, refractive error greater than ±6 dpt and acute ON or treatment with corticosteroids within three months prior to OCT examination.

### Ethics Statement

The study was approved by the local ethics committee at the Charité - Universitätsmedizin Berlin and was conducted following the Declaration of Helsinki in its currently applicable version, the guidelines of the International Conference on Harmonisation of Good Clinical Practice and the applicable German laws. All participants gave informed written consent.

### Optical Coherence Tomography

**Figure 1:**
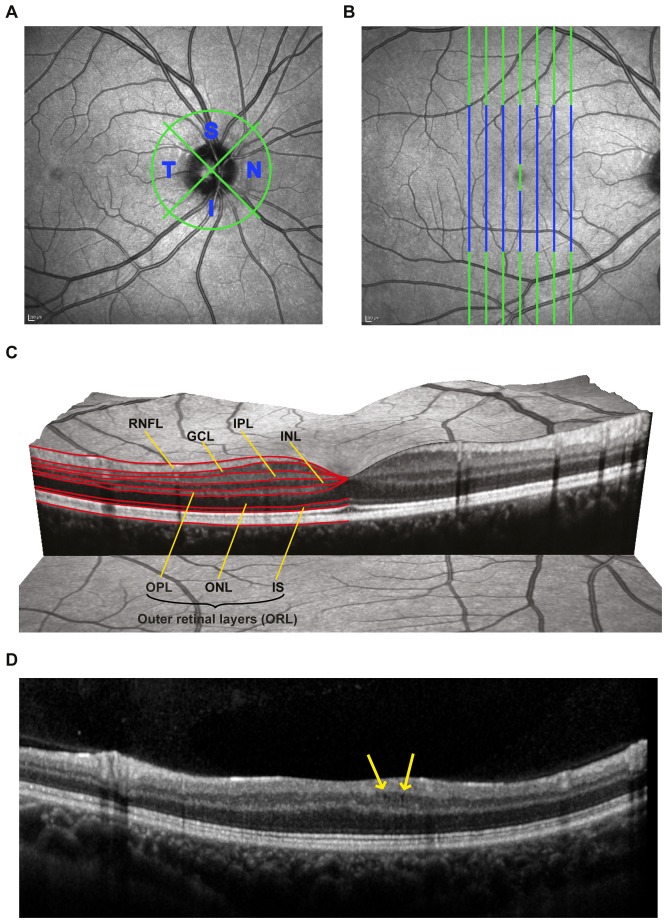
Sample OCT measurement and segmentation. A) Sample scanning laser ophthalmoscopy image showing the peripapillary ring-scan for retinal nerve fiber layer analysis. Nasal and temporal quadrants were analyzed separately B) Sample scanning laser ophthalmoscopy image showing the B-scans included in the segmentation procedure (green and blue) and the area included into analysis (blue only). C) Sample macular scan showing the segmentation lines and intra-retinal layer layout. Red segmentation lines provided by the software define the macular retinal nerve fiber layer (mRNFL), the ganglion cell layer (GCL), the inner plexiform layer (IPL), the inner nuclear layer (INL), the outer plexiform layer (OPL), the outer nuclear layer (ONL), and inner segments of the photoreceptor layer (IS). OPL, ONL and IS were analyzed combined as outer retinal layers (ORL). D) Sample B-scan of an NMOSD patient with microcystic macular edema (MME).

OCT examination was performed with the Spectralis SD-OCT (Heidelberg Engineering, Heidelberg, Germany) without pupil dilatation. RNFL thickness was measured using a 3.4 mm circular scan around the optic nerve head with the device’s standard protocol and segmentation algorithm with activated eye tracker. Ring scan parameters included into analysis were average peripapillary RNFL (pRNFL), quadrant RNFL thicknesses (figure 1A), and nasal to temporal RNFL ratio (N/T ratio). The TMV was assessed by a custom scan that uses 61 vertical B-scans (each with 768 A-Scans, ART = 13 frames) with a scanning angle of 30°×25° focusing on the fovea. All scans passed published quality control criteria [45].

### Intra-retinal Layer Segmentation

Heidelberg Engineering provided a beta software version with a multilayer segmentation algorithm for macular volume scans (Spectralis software version 5.5.0.5, Eye Explorer Software 1.7.0.0). To analyze the inner retinal layers of macular volume scans a subset of B-scans were segmented and manually corrected by an experienced rater in a blinded fashion. The multilayer analysis was performed on the central B-scan through the fovea and on three B-scans each in nasal and temporal direction. Every fourth B-scan from the original volume scan was used resulting in a distance between analyzed B-scans of approximately 500 µm. The mean thickness was calculated for the following retinal layers (figure 1C): macular RNFL (mRNFL), GCL, inner plexiform layer (IPL) and inner nuclear layer (INL). The outer retinal layers (ORL), including the outer plexiform layer (OPL), outer nuclear layer (ONL) and inner photoreceptor layer segments (IS), were analyzed in combination. We analyzed thickness data within a box centered around the fovea and excluding the superior and inferior parts of the scan prone to vessel artifacts as well as the central fovea (Figure 1B). Microcystic macular edema (MME) was defined as presence of cystic lesions located in the inner nuclear layer on at least two adjacent B-scans [46].

### Visual Function Testing

High contrast visual acuity (VA) of both eyes was assessed monocularly for both eyes using ETDRS charts integrated in the Optec 6500 P system (Stereo Optical Co, Inc, Illinois, USA) on the same day as OCT examination in all groups. The resulting value was converted into Snellen equivalents. Low contrast visual acuity (LCVA) was assessed using Functional Acuity Contrast Testing with the Optec 6500 P system. Both eyes were tested in monocular mode following the standards published by the American National Standards Institute with best correction under photopic (“day light” with target luminance value of 85 cd/m^2^) conditions without glare as previously described in detail [47]. Briefly, the linear sine-wave grating charts tested for five spatial frequencies, each with nine levels of contrast. Contrast sensitivity was then recorded as the lowest contrast level achieved by a patient for each spatial frequency. From the five spatial frequency measurements, a function over all measurements was calculated using a least square curve fit and the area under the curve was then established as the area under this function between the lowest and highest spatial frequency, providing a summary expression over all measurements.

### Statistical Analysis

Cohort differences were analyzed using Kruskal Wallis test for age of all groups and Mann-Whitney-U tests (MWU) for time since diagnosis and time since last ON of the NMOSD and the MS group. To investigate differences in OCT measures between the different cohorts and between ON and NON eyes, generalized estimation equation models (GEE) with working correlation matrix ‘exchangeable’ were used accounting for within-patient inter-eye dependencies. In all GEE models, diagnosis was used as independent variables and OCT parameters as dependent variables. To account even for potential effects of demographic variables we also corrected for age and gender. The monocular association between GCL and visual function was tested using linear regression models (LR) with GCL as independent and visual function as dependent variable with no further covariates. For subgroup analyses (ON or NON eyes) only the respectively matched HC eyes were included in the analyses, resulting in different, but exactly eye-wise matched cohorts.

All statistical analyses were performed with SPSS 20 (IBM SPSS, NY, USA), all graphical figures were created using R (basic R version 2.15.2 including the ggplot2 package). Statistical significance was achieved at *p*<0.05. All tests should be understood and interpreted as constituting exploratory data analysis in such way that no previous power calculation or adjustments for multiple testing were made.

## Results

The demographic data of the study participants is summarized in table 1. Complete data was available from all patients for OCT and high contrast VA. In addition, LCVA was available from 13 NMOSD patients. To avoid inclusion bias only the LCVA results of the respectively matching 13 MS patients were included. Six NMOSD and 6 MS patients had a history of ON for both eyes, eight patients in each group had a unilateral ON and three patients had LETM without history of ON. There were no significant differences in age between the cohorts (Kruskal Wallis test, p = 0.893), yet disease duration was significantly longer in the MS group compared to the NMOSD group (MWU, p = 0.026). There was no significant difference in time since last ON between MS and NMOSD patients’ eyes with a history of ON (MWU, p = 0.181).

**Table 1:** Demographic and clinical overview.

		NMOSD	RRMS	HC
**Subjects**	*n*	17	17	17
**Gender**	Female	16	16	16
	Male	1	1	1
**Age [years]**	mean ± SD	40.8±12.3	41.2±12.7	41.4±12.4
	Min–Max	19–63	20–64	21–66
**Time since diagnosis [months]**	Mean ± SD	44.3±38.2	93.7±65.8	n/a
	Min–Max	3–129	3–240	n/a
**AQP4-Ig positive**	*n*	16	n/a	n/a
**Eyes**	*n*	34	34	34
**Eyes with a history** **of ON**	*n*	20	20	n/a
**Time since last ON [months]**	Mean ± SD	45±41	65±46	n/a
	Min–Max	3–130	3–160	n/a

**Abbreviations:** NMOSD: neuromyelitis optica spectrum disorders; RRMS: relapsing-remitting multiple sclerosis; HC: healthy controls; AQP4-Ig: aquaporin 4 antibodies; ON: optic neuritis, n/a = not applicable (the resp. data did not apply to this group).

### OCT Measures in NMOSD and MS Eyes with ON

**Figure 2:**
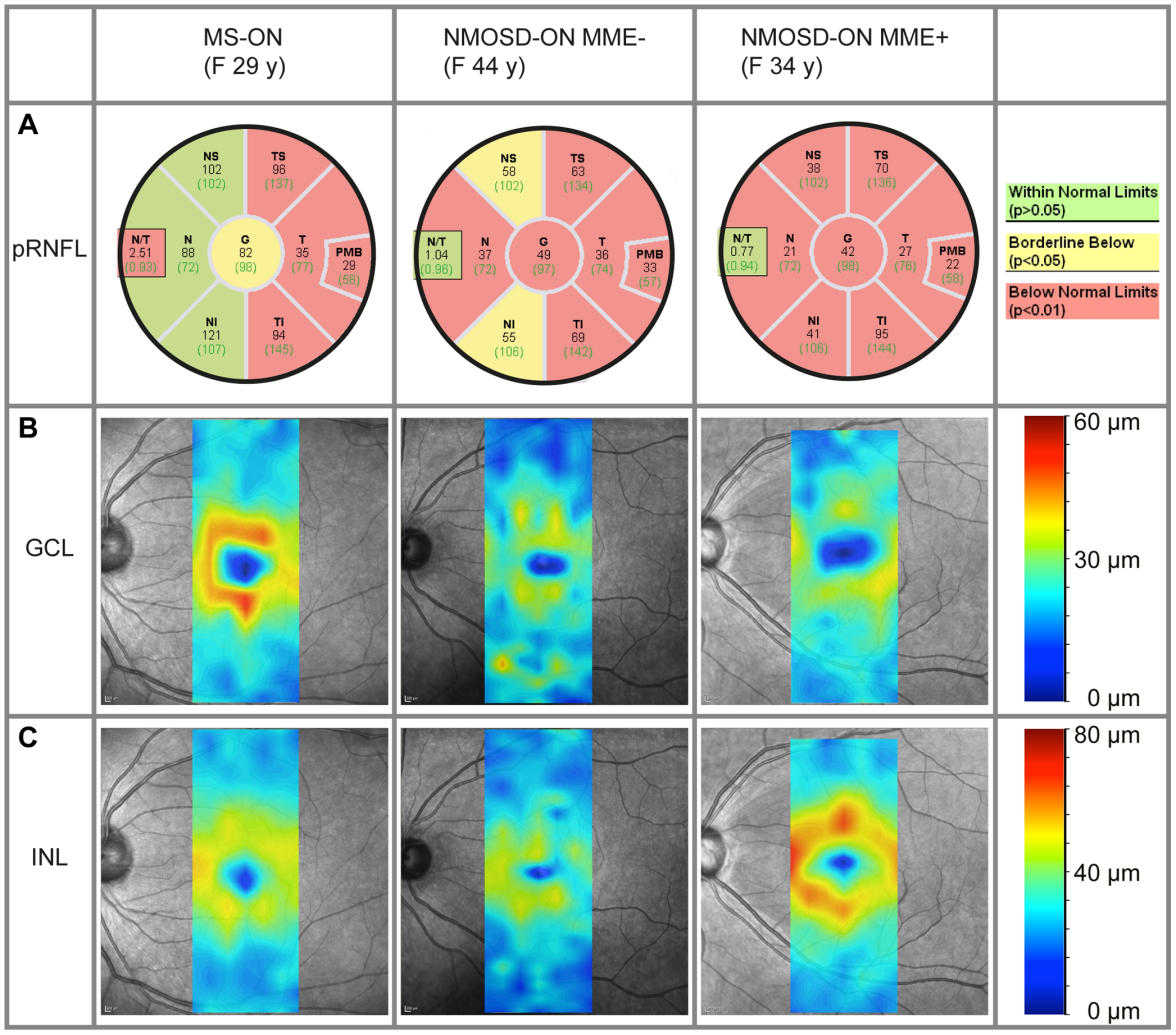
Sample patient data from NMOSD and MS eyes. A) Peripapillary retinal nerve fiber layer (pRNFL) thickness data (in µm) for average RNFL (G) and sectors (nasal-superior quadrant (NS), temporal-superior (TS), temporal, temporal-inferior (TI), nasal-inferior (NI) and nasal (N)) for a multiple sclerosis (MS) patient’s eye with a previous optic neuritis (ON) (left), a neuromyelitis optica spectrum disorder (NMOSD) patient’s eye with a previous ON without microcystic macular edema (MME) (center), and an NMOSD patient’s eye with previous ON and MME (right). Background colors describe the comparison to a healthy reference group from the device’s database. B) and C) Thickness maps of the retinal ganglion cell layer (GCL, B) and inner nuclear layer (INL, C) respective to the patients’ data from A).

mRNFL, GCL and IPL were significantly reduced in NMOSD-ON eyes compared to HC (table 2). On the contrary, INL thickness and the outer retinal layers were significantly increased in NMOSD-ON eyes when compared to HC eyes. NMOSD-ON eyes also showed a significantly more pronounced mRNFL, GCL and IPL thinning than MS-ON eyes while slight differences in INL and the outer retinal layers were not significant (sample cases in figure 2).

**Table 2:** Retinal morphology in eyes after ON.

	NMOSD-ON		MS-ON (vs. NMOSD-ON)				Matched HC (vs. NMOSD-ON)			
	Mean ± SD	Min–Max	Mean ± SD	Min–Max	B (SE)	*p*	Mean ± SD	Min–Max	B (SE)	*p*
**Average pRNFL (µm)**	58.5±21.2	28.0–93.4	85.3±13.3	62.3–105.2	−**26.7 (5.3)**	**<0.001**	100.1±10.8	80.7–122.3	**41.5 (5.1)**	**<0.001**
**inferior pRNFL (µm)**	79.18±29.3	36.1–134.9	115.4–17.9	81.1–144.9	−**35.9 (7.1)**	**<0.001**	131.0±14.9	97.4–155.0	−**51.6 (7.1)**	**<0.001**
**superior pRNFL (µm)**	73.6±26.1	25.2–124.9	105.7±18.4	71.8–138.0	−**31.9 (7.1)**	**<0.001**	120.6±16.0	94.0–157.1	−**46.7 (7.2)**	**<0.001**
**nasal pRNFL (µm)**	38.6±19.5	11.3–87.8	67.7±15.1	37.8–89.0	−**29.1 (5.4)**	**<0.001**	76.5±19.7	35.5–105.0	−**37.8 (5.9)**	**<0.001**
**temporal pRNFL (µm)**	42.4.±16.6	17.9–78.7	52.5±14.7	35.1–89.8	−**9.9 (4.6)**	**0.031**	72.2±10.4	53.2–95.6	−**29.7 (4.3)**	**<0.001**
**TMV (mm^3^)**	7.96±0.47	7.04–8.67	8.31±0.48	7.44–9.02	−**0.34 (0.14)**	**0.019**	8.52±0.53	7.75–9.38	−**0.56 (0.15)**	**<0.001**
**mRNFL (µm)**	25.6±6.2	13.2–35.5	29.4±4.0	22.8–37.5	−**3.7 (1.5)**	**0.015**	33.4±2.3	29.5–36.4	−**7.8** **(1.4)**	**<0.001**
**GCL (µm)**	30±8.5	9.1–46.3	36.1±5.8	27.1–44.4	−**6.1 (2.2)**	**0.007**	45.5±5.1	37.8–56.3	−**15.4 (2.1)**	**<0.001**
**IPL (µm)**	26.0–7.0	16.4–38.2	33.1±7.0	22.5–44.8	−**7.0 (2.1)**	**<0.001**	35.9±5.2	27.6–45.5	−**9.8 (1.9)**	**<0.001**
**INL (µm)**	41.5±5.3	34.5–54.1	39.3±3.1	33.4–44.6		0.075	38.9±3.1	33.2–45.9	**2.6 (1.3)**	**0.045**
**Outer layers (µm)**	114.6±6.6	96.7–128.5	113.5±9.6	102.0–134.8		0.683	110.3±6.94	99.2–122.9	**4.2 (2.0)**	**0.035**

Results of retinal OCT outcomes of NMOSD and MS patients’ eyes after optic neuritis and healthy controls; GEE results showing the differences between NMOSD-ON and MS-ON and NMOSD-ON to healthy controls.

**Abbreviations:** NMOSD: neuromyelitis optica spectrum disorder; MS: multiple sclerosis; NON: eyes without history of optic neuritis; HC: healthy controls; OCT: optical coherence tomography; p/mRNFL: peripapillary and macular retinal nerve fiber layer; TMV: total macular volume; GCL: ganglion cell layer; IPL: inner plexiform layer; INL: inner nuclear layer; SD: standard deviation; Min: minimum; Max: maximum; B: coefficient estimate from generalized estimating equation models (GEE), SE: standard error from GEE coefficient estimates.

**Figure 3:**
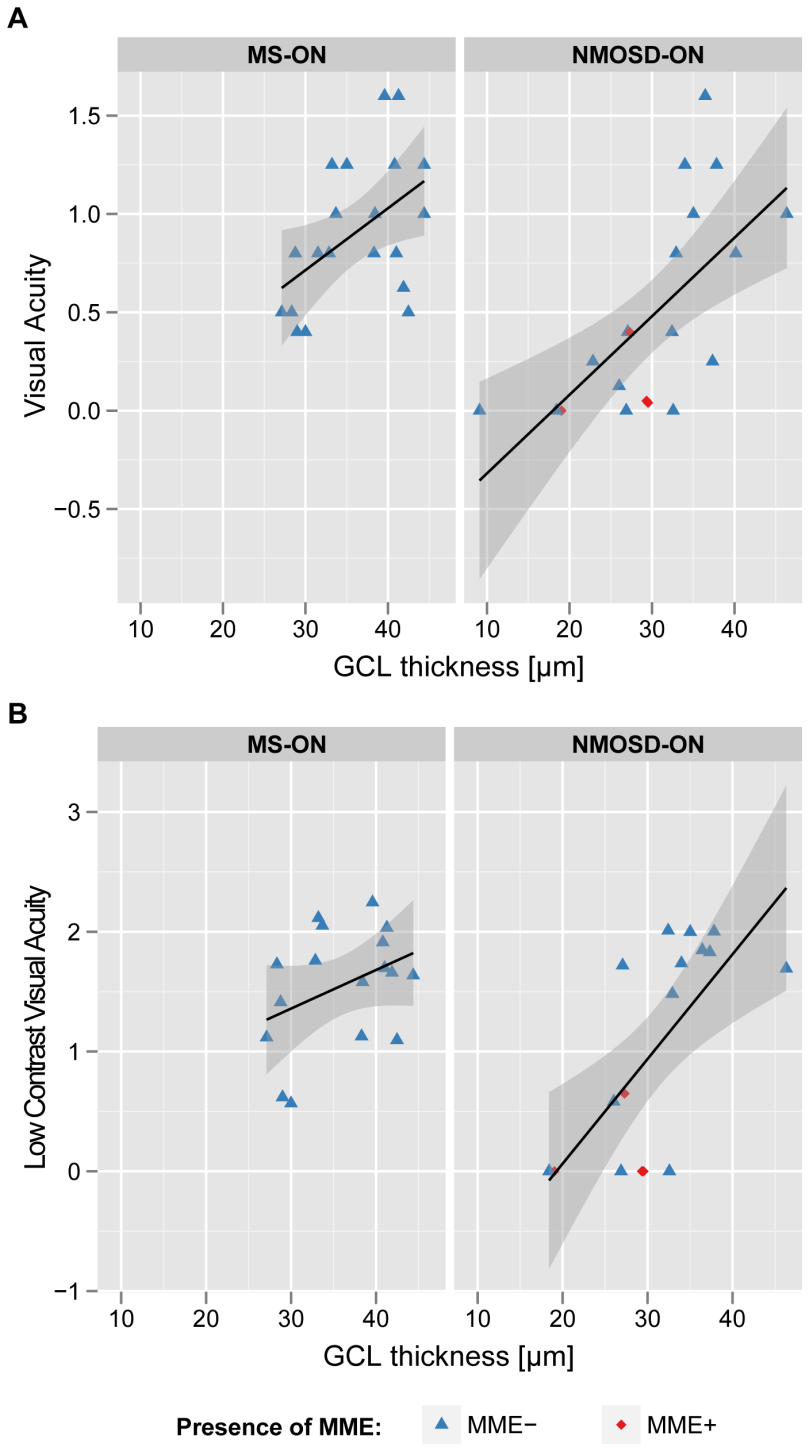
Correlation between visual function and retinal morphology. Scatterplots illustrating relations of ganglion cell thickness of neuromyelitis optica spectrum disorder (NMOSD) and multiple sclerosis (MS) patients’ eyes with a previous optic neuritis to A) high contrast visual acuity (determined by ETDRS charts) and B) low contrast visual acuity determined by functional acuity contrast testing.

ON in NMOSD eyes resulted in stronger visual function impairment than ON in MS eyes: High contrast VA was significantly lower with NMOSD-ON eyes (Mean ± SD: 0.48±0.51) compared to MS-ON eyes (0.91±0.37, GEE: B = −0.4, SE = 0.1, p = 0.002). Likewise, low contrast LCVA was reduced with NMOSD-ON eyes (1.03±0.89) when compared to MS-ON eyes (1.55±0.50, GEE: B = −0.5, SE = 0.2, p = 0.024). GCL thickness was related to VA in NMOSD-ON eyes (LR: R^2^ = 0.441, p = 0.001) better than in MS-ON eyes (LR: R^2^ = 0.242, p = 0.028) (figure 3A). The same held true for LCVA in NMOSD-ON (LR: R^2^ = 0.467, p = 0.002) versus MS-ON eyes (LR: R^2^ = 0.143, p = 0.135) (figure 3B).

### Different Affection of Quadrant pRNFL in NMOSD and MS after ON

pRNFL thickness was significantly decreased in eyes from patients with either of the two disorders compared to HC, with NMOSD-ON eyes being significantly more affected than MS-ON eyes in all quadrants (table 2). As it is a long-standing clinical experience that optic disc pallor and optic atrophy in MS-ON eyes may exhibit a temporal preponderance and as also histopathological and OCT works have shown particular damage to temporal axons [48]–[50], we were curious whether a predilection of the temporal quadrant of pRNFL would also be detectable in NMOSD. While pRNFL was indeed primarily reduced in the temporal quadrant in MS-ON eyes, pRNFL in NMOSD-ON eyes was more broadly reduced with thinning involving other quadrants. We used the ratio of nasal versus temporal RNFL thickness (N/T ratio) for comparing quadrant-wise thinning in the groups NMOSD-ON and MS-ON. N/T ratio was significantly lower in NMOSD-ON than in MS-ON (0.93±0.35 vs. 1.36±0.43; GEE: B = −0.38, SE = 0.13, p = 0.004), meaning that in relation to the temporal quadrant, the nasal quadrant was much stronger affected in NMOSD than in MS. These results are emphasized by the representative pRNFL quadrant thickness values in figure 2.

To further analyze the potential value for differential diagnosis we calculated ROC curves. Here, pRNFL showed an AUC of 0.835 (corresponding to 60% sensitivity at 90% specificity to identify an NMOSD related optic neuritis by this measure) and N/T ratio an AUC of 0.775 (corresponding to 50% sensitivity at 90% specificity). These values should be interpreted with caution due to the low number of cases and the exploratory nature of the analysis and are only given for comparison of our results to other studies.

To investigate whether the N/T ration may be NMOSD specific or whether a severe ON might lead in general to a broader loss of pRNFL, we selected 20 additional eyes with a history of ON from 17 RRMS patients presenting with very thin pRNFL values from our OCT database. In order to analyze MS patients with the most severe pRNFL reduction, we simply chose those 17 RRMS patients in our database with the lowest pRNFL values without any further criteria or a specific cutoff. Despite these eyes (59.2±6.0 µm) showing a comparable pRNFL thickness with NMOSD-ON eyes (58.5±21.2 µm, p = 0.984), the N/T ratio in these eyes (1.47±0.42) was in the range of the originally matched MS-ON eyes (GEE: p = 0.361) and statistically highly significantly different from NMOSD-ON eyes (GEE: B = −0.53, SE = 0.13, p<0.001), further supporting the evidence for a different damage pattern in NMOSD compared to MS.

None of the MS-ON eyes showed a pRNFL below 46.6 µm whereas 9 NMOSD-ON eyes (45%) had a pRNFL below this value. No MS-ON eyes showed an N/T ratio below 0.61, while 5 NMOSD-ON eyes (25%) were below this limit. Four NMOSD-ON eyes (20%) showed both pRNFL and N/T ratio below these cut-off values.

### Role of Microcystic Macular Edema (MME)

**Figure 4:**
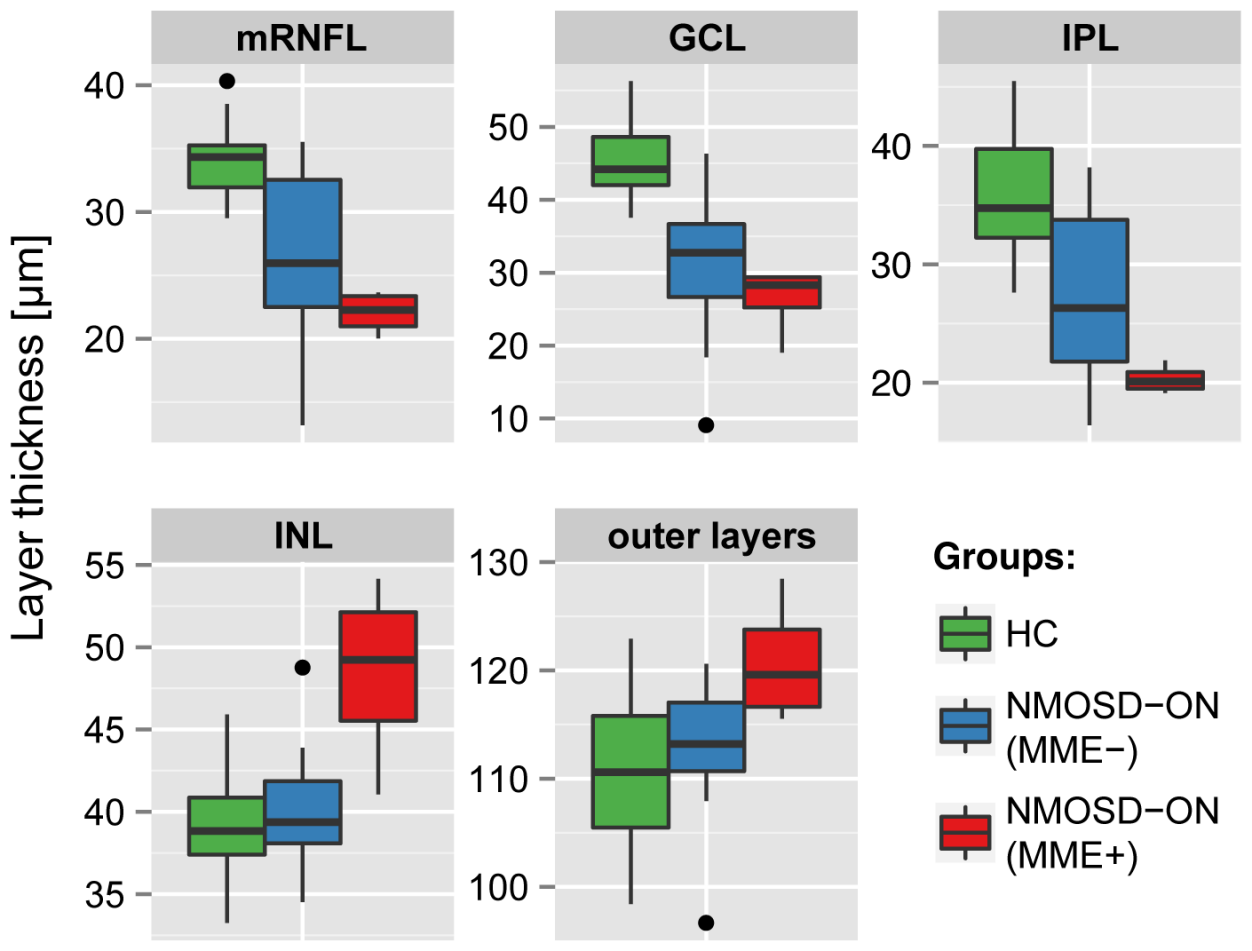
Intra-retinal layer thickness in NMOSD-ON eyes with and without microcystic macular edema. Layer thicknesses for the macular retinal nerve fiber layer (mRNFL), ganglion cell layer (GCL), inner plexiform layer (IPL), inner nuclear layer (INL) and the combined outer retinal layers for NMOSD-ON eyes with (MME+, in red) and without (MME-, in blue) microcystic macular edema and healthy controls eyes (HC, in green). Outer retinal layers include outer plexiform layer, outer nuclear layer and inner photoreceptor layer segments.

Four eyes from three NMOSD patients (75%) were affected by MME (figure 1D). All patients with MME reported a previous ON in the respective eye. In contrast, no MS patient or HC was affected by MME. Eyes with MME (MME+) in NMOSD patients showed severely reduced RNFL, GCL, and IPL, but increased INL and outer layers in comparison to NMOSD-ON eyes without MME (MME-) (figure 4, MME+ in red).

All MME+ eyes performed very poorly in high and low visual acuity testing with three eyes being legally blind. VA was significantly lower in MME+ eyes (Mean ± SD 0.12±0.19) than in MME- eyes (0.57±0.53, GEE: B = 0.609, SE = 0.123, p<0.001) and LCVA was reduced in MME+ eyes (0.16±0.33) when compared to MME- eyes (1.30±0.83, GEE: B = 1.237, SE = 0.163, p<0.001).

To test whether the above reported INL and outer layer thickening in NMOSD-ON eyes over HC eyes was a result of MME, we performed all corresponding group comparisons also under exclusion of MME affected eyes and their respective controls. Comparing these cohorts, no significant increase of INL (p = 0.363) and outer layers (p = 0.336) was detected in NMOSD eyes anymore. pRNFL (B = −38.6, SE = 5.8) and the inner retinal layers mRNFL (B = −6.9; SE = 1.7), GCL (B = −15.2, SE = 2.6) and IPL (B = −8.7, SE = 2.0) were still significantly decreased in comparison to HC eyes (all p<0.001). These layers were also reduced in NMOSD-ON eyes compared to MS-ON eyes (mRNFL: B = - 3.7, SE = 1.5, p = 0.015; GCL: B = −6.1, SE = 2.2, p = 0.007; IPL: B = −7.0, SE = 2.1, p = 0.001).

### NMOSD vs. MS Eyes without Previous Optic Neuritis

Finally, we investigated whether NMOSD patient’s eyes without any history of ON showed retinal changes in comparison to healthy controls. Detailed results comparing retinal measures of NMOSD-NON with eyes from HC are given in table 3. In summary, NMOSD-NON eyes did not differ significantly from HC eyes in any of the OCT layer measures. For comparison with MS, we additionally compared MS-NON eyes with HC. MS-NON eyes showed reduced thickness in macular RNFL and GCL and a slight non-significant thickening of INL and outer layers.

**Table 3:** Retinal morphology in eyes without previous ON.

	Matched HC		NMOSD-NON (vs. HC)			MS-NON (vs. HC)			
	Mean ± SD	Min–Max	Mean ± SD	Min–Max	*p*	Mean ± SD	Min–Max	B (SE)	*p*
**Average pRNFL (µm)**	97.0±12.1	80.4–120.8	99.5±7.5	88.9–113.7	0.5	92.5±8.9	71.5–100.9		0.69
**TMV (mm^3^)**	8.52±0.53	7.75–9.38	8.45±0.34	8.02–9.16	0.355	8.59±0.28	8.21–9.13		0.086
**mRNFL (µm)**	34.4±2.9	26.6–40.3	34.8±2.0	32.4–38.4	0.61	31.7±2.9	27.0–39.4	−**2.4 (1.1)**	**0.025**
**GCL (µm)**	44.9±4.2	37.5–52.8	43.3±2.0	36.6–50.5	0.293	41.3±5.3	31.5–49.0	−**3.6 (1.7)**	**0.039**
**IPL (µm)**	35.8±3.8	30.4–44.3	35.3±4.4	26.9–40.9	0.69	34.4±3.5	29.0–39.0		0.266
**INL (µm)**	39.1±2.4	33.8–43.8	38.4±3.4	32.3–46.7	0.507	40.5±2.2	36.3–43.7		0.095
**Outer layers (µm)**	110.5±6.5	98.4–122.9	109.3±5.4	94.7–116.3	0.52	113.3±5.4	103.1–123.9		0.201

Results of retinal OCT outcomes of NMOSD and MS patients’ eyes without history of optic neuritis and healthy controls; GEE results showing the differences between NMOSD-NON and MS-NON to healthy controls. Outer retinal layers include outer plexiform layer, outer nuclear layer and inner photoreceptor layer segments.

**Abbreviations:** NMOSD: neuromyelitis optica spectrum disorder; MS: multiple sclerosis; NON: eyes without history of optic neuritis; HC: healthy controls; OCT: optical coherence tomography; p/mRNFL: peripapillary and macular retinal nerve fiber layer; TMV: total macular volume; GCL: ganglion cell layer; IPL: inner plexiform layer; INL: inner nuclear layer; SD: standard deviation; Min: minimum; Max: maximum; B: coefficient estimate from generalized estimating equation models (GEE), SE: standard error from GEE coefficient estimates.

## Discussion

In this study we used SD-OCT with intra-retinal segmentation to investigate retinal layer changes in 17 NMOSD patients in comparison to matched RRMS patients and HC. In line with clinical data and previous OCT studies [32], [36], [37] we show that neuroaxonal retinal damage displayed by thinning of the peripapillary and macular RNFL and of the GCL is more severe in ON eyes from patients with NMOSD than in ON eyes from patients with MS. This is paralleled by poorer visual functions as assessed by high and low contrast visual acuity. Interestingly, the association of structural retinal damage and impairment of visual function was more apparent in NMOSD-ON eyes than in MS-ON eyes: GCL thickness was a much stronger predictor of both ETDRS visual acuity and LCVA measured by Functional Acuity Contrast Testing in NMOSD than in MS. Half of NMOSD-ON eyes had pRNFL values below 46.6 µm versus none of the MS-ON eyes, and the mean pRNFL difference between both groups was 27 µm. This difference is in striking accordance with values reported by two previous seminal studies in NMO using the older time domain OCT technology [29], [30]. The stronger association between morphology and visual function in NMOSD-ON eyes that tend to have lower axonal and neuronal OCT measures may suggest that below a certain threshold of neuroaxonal loss retinal neurons and axons are no longer able to sufficiently maintain visual function. Other studies in patients with ON or NMO further underscore the assumption of a threshold RNFL thickness, below which visual acuity becomes very poor [30], [51], [52]. This has important implications as early and effective therapeutic interventions following ON should aim to prevent substantial retinal damage and thus poor visual function which negatively influences the patient’s quality of life [53], [54]. Several recent studies have indeed shown that therapeutic interventions with corticosteroids, plasma exchange or erythropoietin after an ON attack may help preserve retinal axons and this effect can be monitored by OCT [28], [55], [56]. This further supports the argument of some authors that strict immunosuppression is mandatory in NMO [57]. Importantly, non-affected eyes in NMOSD had normal visual function and preserved retinal layers.

Our results support previous findings on a distinct distribution of RNFL thinning across quadrants in NMOSD-ON eyes versus MS-ON eyes. Consistent with Naismith et al. [29] and Monteiro et al. [32], the temporal preponderance of RNFL damage typical for MS was not detectable in NMOSD eyes. Here, other quadrants were as well severely affected, resulting in a significantly lower nasal to temporal pRNFL ratio in NMOSD versus MS. However, given the limited sample size in our and the two previous cohorts and the substantial overlap of data between MS and NMOSD, the N/T ratio is currently of limited value to differentiate between a NMOSD-ON and a MS-ON eye on an individual level. The pathophysiological aspects, however, deserve discussion. The temporal quadrant of the RNFL contains the papillo-macular bundle, which is built by parvocellular axons that consist of smaller, thinly myelinated fibers with rapid firing rates. Interestingly, several diseases besides MS show a predominant impairment of these parvocellular axons such as Leber’s hereditary optic neuropathy, OPA1 related dominant optic nerve atrophy, and spinocerebellar ataxia type 1 [58]. All these diseases share a presumed insult to mitochondria as one key event in the disease process. Owing to their small volume and fast firing rates, parvocellular axons may be more vulnerable to energy depletion resulting from impaired mitochondrial function [59].

Support for this assumption stems from an autopsy study by Evangelou et al. who demonstrated a selective vulnerability to injury of parvocellular axons and neurons in the anterior optic pathway in MS [60]. In contrast, the more even distribution of retinal damage in NMOSD-ON eyes as compared to MS-ON may indicate that damage to parvocellular axons is not a key feature in NMO but other mechanisms are more important.

In line with some previous reports [30], [33], NMOSD-NON eyes exhibited no apparent retinal damage as all OCT measures were not different from controls. This is concordant with the notion that retinal damage in NMO is linked to clinically manifest ON attacks and does not occur progressively or as a consequence of subclinical optic neuropathy as is the case in MS. Accordingly, a secondary progressive course has been rarely described in NMO [35]. In contrast to our and other groups Syc et al. and Sotirchos et al. reported GCL plus IPL thinning also in NMOSD eyes without history of ON [36], [39]. As the authors acknowledge, subclinical disease activity in NMOSD is not a widely appreciated phenomenon. These discrepant findings from studies using high resolution SD-OCT with intra-retinal segmentation can currently not be resolved; the use of two different OCT devices and segmentation techniques and the relatively low number of NMOSD-NON eyes in both studies with an inherent risk for statistical errors may have played a role. However, the topic deserves to be addressed in larger studies, as the finding of subclinical optic neuropathy in NMO would question our current pathophysiological understanding of NMO.

Of note are our findings of MME in four NMOSD-ON eyes. MME in these few eyes was responsible for the apparent thickening of the INL in NMOSD-ON eyes versus HC that was no longer present when we excluded these MME eyes from group comparisons. MME has recently been described by Gelfand and colleagues in 15 of 318 MS patients [46]. MME was associated with disease severity, reduced visual acuity and RNFL thinning. Moreover, MME was predominantly located in the INL and was more prevalent in eyes with prior symptomatic ON. However, the question as to whether MME is specific for MS or may also occur in other conditions with optic nerve involvement remains to be answered [61], [62].

In line with this, both Sotirchos et al. and Gelfand et al. [38], [39] reported MME exclusively in NMO eyes affected by ON as was the case in our study. Moreover, all three works consistently found that MME NMO eyes exhibited more severe structural retinal damage and more profoundly impaired visual function than non MME NMO eyes. In this regard, the recent proposal by Balk and colleagues that MME may be linked to Mueller cell pathology is intriguing and warrants further investigation [61]. In NMO one could hypothesize targeting of Mueller cells which are AQP4-containing retinal astrocytes of the retina by AQP4 autoantibodies. In AQP4 knock-out mice, electroretinograms have suggested that lack of AQP4 mildly impairs retinal function, presumably by altered Mueller cell fluid balance [63]. However, it remains to be investigated if AQP4 antibodies that have been shown to be pathogenic in NMO [64], may have access to the retina via a leaky blood-retina barrier and thus can target retinal Mueller cells. This could be another cause of retinal damage beyond a retrograde degeneration of ganglion cells following an attack to axons in the optic nerve [36].

In light of recent studies that have shown a detrimental effect of some MS disease-modifying drugs in NMO [65]–[73], the possibility of using OCT for a more accurate diagnosis of NMO and for a correct differential diagnosis versus MS would be desirable, especially in AQP4 antibody negative patients. However, despite some distinct features of retinal damage we and others have identified by OCT group comparisons, the discriminatory capacity of OCT for a reliable differential diagnosis of the individual patient is still insufficient to be used in clinical routine. Moreover, it is a limitation of our and most previous studies that the sample size of the NMO cohorts were relatively small owing to the rarity of NMO, thus results are prone to statistical errors. Further technical advances in OCT technology with respect to data acquisition and post-processing will hopefully improve the utility of the technique for clinically routine in the near future.
